# Protocol for a proof of concept randomized controlled trial of cognitive-behavioural therapy for adult ADHD as a supplement to treatment as usual, compared with treatment as usual alone

**DOI:** 10.1186/s12888-014-0248-1

**Published:** 2014-09-03

**Authors:** Antonia J Dittner, Katharine A Rimes, Ailsa J Russell, Trudie Chalder

**Affiliations:** King’s College London, King’s Health Partners, Behavioural and Developmental Psychiatry Clinical Academic Group, Maudsley Adult ADHD Service, South London and Maudsley NHS Foundation Trust, London, UK; King’s College London, King’s Health Partners, Department of Psychology, Institute of Psychiatry, Psychology and Neuroscience, London, UK; King’s College London, Department of Psychology, Institute of Psychiatry, Psychology and Neuroscience, London, UK and Department of Psychology, University of Bath, Bath, UK; King’s College London, King’s Health Partners, Department of Psychological Medicine, Institute of Psychiatry, Psychology and Neuroscience, London, UK

**Keywords:** Adult attention deficit hyperactivity disorder, Adult ADHD, Cognitive behavioural therapy, Randomized controlled trial, Psychosocial treatment, Psychological treatment, Psychotherapy

## Abstract

**Background:**

ADHD is prevalent in adults and frequently associated with impairment and distress. While medication is often the first line of treatment a high proportion of people with the condition are not fully treated by medication alone, cannot tolerate medication or do not wish to take it. Preliminary studies suggest that psychosocial approaches are a promising adjunctive or alternative treatment option. To date, individual cognitive-behaviour therapy (CBT) has been found to be efficacious in three randomized controlled trials (RCTs). There is a need for more RCTs to be carried out in order to replicate these results in different sites, to further investigate the acceptability and feasibility of CBT in this population and to further develop CBT approaches based on a psychological model. This randomized controlled trial investigates the efficacy of individual, formulation-based CBT when added to treatment-as-usual as compared with treatment as usual alone.

**Methods/design:**

Sixty patients with a diagnosis of adult ADHD attending a specialist clinic are randomly allocated to 1 of 2 treatments, ‘Treatment as Usual’ (TAU) or TAU plus 16 sessions individual CBT (TAU + CBT). In the TAU + CBT, the first 15 sessions take place over 30 weeks with a 16th ‘follow-up’ session at 42 weeks. Outcomes are assessed at 30 weeks and 42 weeks following randomization. The two primary outcomes are self-rated ADHD symptoms and functioning (occupational and social). Secondary outcomes include distress, mood, ADHD-related cognitions, ADHD-related behaviours and informant-rated ADHD symptoms.

The primary analysis will include all participants for whom data is available and will use longitudinal regression models to compare treatments. Secondary outcomes will be analysed similarly.

**Discussion:**

The results of the study will provide information about a) whether CBT adds benefit over and above TAU for ADHD and, b) if CBT is found to be efficacious, potential mechanisms of change and predictors of efficacy.

**Trial registration:**

Current Controlled Trials ISRCTN03732556, assigned 04/11/2010

## Background

### Introduction

Although Attention Deficit Hyperactivity Disorder (ADHD) was originally identified in children, it is increasingly recognised that symptoms may persist into adulthood. The disorder is frequently associated with impairment, distress and psychiatric co-morbidity [1,2]. Estimates of prevalence range from 2.5% to 4% cross-nationally [3].

Medication is the first line treatment for ADHD in adults. However, approximately 50% of individuals with adult ADHD are not able to tolerate, do not respond to, or fail to reach optimal outcomes on medication alone [4]. The NICE guidelines for adult ADHD, released in September 2008, recommend that medication be complemented by group and individual CBT and emphasise the need for further research into psychological approaches to treat the condition [5].

There is promising preliminary evidence suggesting that psychological treatment approaches are of benefit for individuals with Adult ADHD [6,7]. A range of short-term, structured therapies, designed to target the specific issues experienced by adults with ADHD have been investigated regarding their impact on self- or independently-rated ADHD symptoms, as well as other outcomes including self-esteem, anger management and mood.

Some of these results are from open-label uncontrolled studies [8-13] which evaluated individual CBT, metacognitive therapy, modified dialectical behavioural therapy (DBT) and mindfulness-based meditation training. All found that following the interventions, participants reported improvements in self- and other-rated ADHD symptoms. There were also improvements in outcomes such as anxiety, depression and functioning.

Other studies have compared potentially efficacious treatments with a control group using a non-randomized design. The studies include group treatments of psychoeducation [14,15]and modified DBT [16]. All found improvements in outcomes in the treatment group (including disorganisation, inattention, emotional lability, knowledge about the condition, self efficacy and self-esteem) compared with the control group. Limitations of all the studies include small sample sizes and the non-randomized designs.

### Randomized controlled trials

There have been seven RCTs of group treatments and these include cognitive remediation programmes [17,18] metacognitive therapy [19] reasoning and rehabilitation [20], DBT [21] and mindfulness based therapies [22,23]. All groups were structured and manualised. Specific skills were taught in each and rehearsal of these skills was encouraged. All studies found statistically significant improvements on the main outcomes in the treatment group compared with the control group following treatment. All showed improvements on self-rated ADHD symptoms while some also demonstrated improvements in independently-rated ADHD symptoms, in objective measures of cognitive functioning and in self-rated associated problems such as low mood and anxiety. Two studies also demonstrated that improvements were maintained at follow-up [18,20].

So far, to the authors’ knowledge, there have been only three RCTs of individual CBT [24-26]. Safren et al. [24] and [25] carried out two RCTs investigating the effectiveness of individual, manualised treatment based on a cognitive behavioural model of ADHD symptoms. Although ADHD specific and using a range of cognitive-behavioural strategies for intervention, the approach was educational and therapist directed rather than individual, formulation driven CBT. For example, information about the role of unhelpful thoughts and coping in general was provided but these were not explicitly linked to the ‘idiosyncratic’ thoughts and behaviours pertinent to each case. Nonetheless, Safren et al., [24] found that participants randomized to CBT and medication as usual (n = 16) had significantly lower clinician rated ADHD symptoms when compared with those randomized to medication alone Large effect sizes (1.2, and 1.7) for between groups change scores were found on clinician and self-rated ADHD symptoms respectively. Those in the CBT group also had significantly lower scores on independently- and self-rated measures of mood. In a subsequent study, Safren et al. [25] compared CBT and medication as usual to a control psychological treatment i.e. relaxation with educational support and medication. Results provided support for greater improvements in the CBT condition rather than relaxation with educational support condition.

Similarly the study by Virta et al. investigated a manualised approach which was not formulation driven, although the topics of a few sessions were chosen by the participants. CBT was compared with cognitive training and no treatment/waitlist conditions. More participants in the CBT condition improved compared with the other two conditions. Both active conditions showed a significant decrease in ADHD symptoms but CBT was superior in efficacy for decreasing ADHD symptoms.

In their 2010 review, Knouse and Safren noted that the treatments with the largest effect sizes commonly comprise short-term, highly structured programmes with an emphasis on teaching specific skills and strategies and practice outside the session [6].

### Rationale for the current study

The psychological treatments developed and evaluated thus far by RCTs have tended to be skills-based i.e. sessions focused on teaching specific skills to combat the symptoms of ADHD such as training in organising and planning. However, the emotional and behavioural problems experienced by adults with ADHD often extend far beyond the impact of the core symptoms themselves.

ADHD in adulthood is the persistence of a childhood condition which has often had a wide-ranging effect on an individual’s emotional, social and cognitive development. For example children with ADHD commonly experience difficulties at school related to their symptoms and may receive negative feedback from parents, teachers and peers. This may lead to the development of negative self-beliefs which persist into adulthood. Consistent with this, it is not uncommon to encounter in adults with ADHD emotional problems such as depression, anxiety and low self-esteem and concurrent maladaptive coping responses [27].

Individual cognitive behavioural therapy is recommended in NICE and other guidelines for ‘common mental health problems’ such as anxiety and depression. At the heart of individual CBT is the development of an individual formulation (the formulation-based approach) in collaboration with the client. Although disorder-specific CBT protocols exist for certain emotional disorders of mild-moderate severity, individual formulation is still important and particularly so for more complex cases or conditions of greater severity.

The formulation is essentially a shared hypothesis as to the relationships between the individual’s predisposition (e.g. cognitive strengths and weaknesses, personality attributes), their experiences, and how these have contributed to the development of certain beliefs, behaviours, emotions and physical reactions. In addition to this developmental aspect, the formulation specifies the possible influence of these beliefs and behaviours on maintaining ADHD symptoms, impairments in daily functioning and distress. Treatment is then tailored to the individual and focuses on the idiosyncratic problematic behaviours and thoughts identified in the detailed assessment, while drawing on a range of evidence-based strategies.

In relation to ADHD, a formulation-based approach may be structured and teach specific skills and strategies, but it would also entail a detailed assessment to identify the main problems experienced by the individual patient which then allows them to be targeted directly. The formulation allows the client and therapist to collaboratively address the core symptoms of ADHD using education, adaptations to the client’s environment and repetition of adaptive skills in order to compensate for executive dysfunction. In addition it allows for the identification and modification of beliefs (e.g. ‘I cannot concentrate so there is no point trying’) and behaviours (e.g. procrastination) that may be serving to maintain problems and associated distress. The therapist can help the client make links between their beliefs and pertinent early life experiences in a compassionate and understanding way and this minimises the possibility of provoking shame or a defensive reaction. This individualised approach improves the client’s understanding of their difficulties and reduces the potential for relapse. This approach would also make it likely that some of the co-morbidities would be addressed during treatment such as anxiety and low mood.

While the RCTs of individual CBT so far have used treatment models, to the authors’ knowledge none has used a formulation-based approach in order to identify and modify idiosyncratic underlying beliefs (conditional assumptions, core beliefs) and compensatory strategies. Formulation-based approaches to ADHD have been described and found to be helpful in previous uncontrolled studies [9,10,12] but this has not been investigated using a randomized controlled design.

The current study therefore aims to develop and evaluate a collaborative, formulation-based approach to treating adult ADHD. A group of individuals receiving CBT combined with treatment as usual for adults with ADHD will be compared with a group receiving treatment as usual only, employing a randomized design. Detailed cognitive-behavioural assessments as well as information about cognitions and behaviours from a measure developed in a concurrent study will inform the content of the intervention. Therapists will also have access to a range of skills-based approaches, as have been used in previous studies, as and when indicated by the formulation, and a treatment manual is being produced which will be updated iteratively.

### Cognitive behavioural process of treatment

#### Mechanisms of change

While common elements of the most effective treatments have been noted, previous studies have not formally investigated mechanisms of change. For example, do symptoms and distress improve because people have learned more effective skills in managing their symptoms? Do negative automatic thoughts about the condition (for example ‘my ADHD will stop me from doing the things I want to do’), underlying beliefs about the self (for example ‘I am a failure’) and associated compensatory strategies need to be changed for CBT to be effective? It is hoped the current study will shed light on the mechanisms of successful CBT for adult ADHD.

#### Predictors of outcome

We will assess demographics, co-morbidity and other clinical factors at baseline to examine whether they are associated with a poorer outcome.

### Aims

To investigate efficacy, patient acceptability and feasibility of a formulation-based cognitive-behavioural therapy for adults with attention deficit hyperactivity disorder (ADHD).

A secondary aim is to investigate the predictors of outcome and mechanisms of change.

### Hypothesis of efficacy

CBT plus treatment as usual will be more effective than treatment as usual alone in i) reducing ADHD symptoms ii) improving functioning.

## Methods/design

### Trial design

A two-arm randomized controlled trial of patients who meet DSM IV criteria for adult ADHD. Participants will be randomly assigned to one of two treatment arms. The first condition is treatment as usual (TAU). TAU will often but not always be pharmacotherapy, overseen either by the ADHD service in which the research is being carried out, the participant’s local adult ADHD Service, a community mental health team or a general practitioner. The second condition is TAU and in addition sixteen sessions of CBT.

NB for brevity, the rest of protocol will refer to the two treatments as CBT and TAU rather than CBT plus TAU and TAU alone.

Each participant will be assessed and participants who give informed consent will be randomly allocated to one of two treatments. Treatment will start as soon as possible after randomization. The final outcome measure will be at 42 weeks after randomization.

### Flow chart of trial design

See Figure 1.

**Figure 1:**
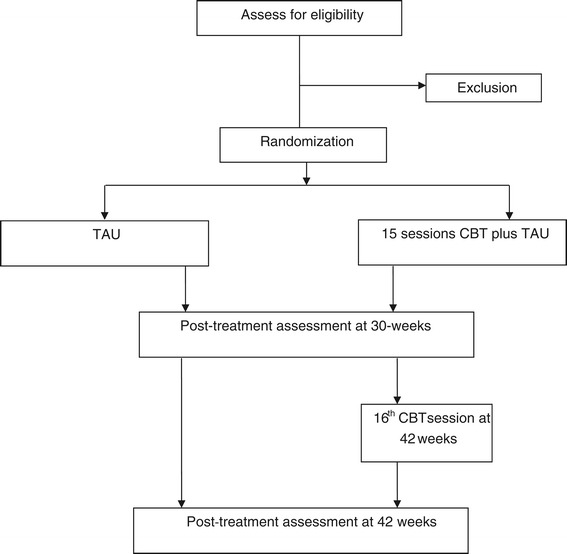
**Flow diagram of trial design.**

### Recruitment

We will study 60 participants over approximately three years. All will have received a diagnosis of adult ADHD, either in the Adult ADHD Service or elsewhere (in this case a copy of the diagnostic report will be required showing external validation of childhood onset). Participants will either already be attending follow-up clinics in the Service, including psychoeducation workshops, or will have been recently referred to the service for medication follow-up or psychological treatment.

The psychoeducation workshops include group discussion, didactic teaching and provision of written materials and cover topics such as what it means to have ADHD, relationships, emotions (anger/frustration, low mood, anxiety) and ADHD, time management, problem-solving, relationships and the future with ADHD. Broadly they follow the format described in a previous study [6] with some adaptations. Results from previous studies suggest that psychoeducation alone, even when developed specifically for ADHD, does not have a significant impact on ADHD symptoms, maybe because it does not incorporate learning and practice of specific skills. It is thought approximately 10-20% of participants will have attended the psychoeducation programme prior to taking part in the trial, however any effects of this are likely to be distributed equally across the two treatment arms owing to the randomized design.

### Inclusion criteria

1. Both clinician and participant agree that randomization is acceptable.
2. The participant has given written informed consent.
3. The participant is aged 18 to 65.
4. The participant is diagnosed with adult ADHD by a mental health professional.
5. The participant’s score on the inattentive or hyperactive/impulsive subscale of the Adult Barkley Current Behaviour Scale (self-rated) is 6 or more.
6. The participant is rated to have clinical severity of at least a moderate level (Clinical Global Impression score of 4 or above).

### Exclusion criteria

1. The research psychologist will use a standardized psychiatric interview (the Mini-International Neuropsychiatric Interview; MINI) under supervision by the PI or nominated deputy to exclude those who have a clinically significant anxiety disorder and current episode of major depression, significant risk of self harm and active substance misuse/dependence in last three months. Participants will also be excluded if they have an acquired brain injury, a primary diagnosis of psychosis or bipolar disorder, a pervasive developmental disorder, a diagnosis of a personality disorder or any other primary clinical diagnosis whereby participation in the trial would be inappropriate to their clinical needs.
2. Participants with a Verbal IQ of less than 80 will be excluded from taking part owing to the verbal and cognitive elements of the intervention.
3. Patients who are considered by the research psychologist in discussion with the PI to be unable to comply with the requirements of an RCT (e.g. are not able to attend regularly and reliably for assessment and treatment sessions), who are currently undergoing another talking therapy for adult ADHD or any other psychiatric disorder, who are unable to speak English at an adequate level to participate in the trial will be excluded.

Eligibility will be established according to the inclusion and exclusion criteria. For pragmatic reasons, to maximize recruitment, it was decided not to have inclusion/exclusion criteria related to the use or otherwise of medication or the stabilisation or otherwise of the dose. Use of medication will be routinely recorded and it is anticipated that any variation in use of medication will be accounted for controlled for in the randomized design.

### Procedures

Individuals likely to meet eligibility criteria for the study will be given an information sheet about the treatment trial by their doctor and invited to take part. Potential participants will attend an assessment appointment with a research psychologist to ascertain eligibility for the study. If the patient understands the purpose of the trial and is willing to give informed consent to be randomized, treated and followed up, they will sign the consent form to participate in the trial.

### Eligibility assessment

A battery of self-report measures and two structured diagnostic interviews will be administered. The Mini-International Neuropsychiatric Interview (M.I.N.I.) assesses for co-morbid psychiatric diagnoses and takes approximately 15 minutes [28]. Conners’ Adult ADHD Diagnostic Interview for DSM-IV (CAADID) aids in diagnosing ADHD in adults [29]. Only the questions pertaining to symptoms in adulthood would need to be administered (since presence of symptoms in childhood would already be established).

### Randomisation and enrolment procedure

Participants will be randomly allocated to one of the two treatments. Randomization will be performed by an independent group in another service in the same NHS Trust using a concealed sequence. The sequence will be generated using randomization tables and fixed-length blocks (length concealed), stratified for gender. The sequence will be generated by one named individual and put into numbered sealed envelopes. The envelopes will be kept by another named individual (the independent researcher) in a locked drawer to which only she has access. When a participant is suitable for randomization the ADHD assistant will email the independent researcher giving the participant number and gender. The independent researcher will reply by email with the allocation and will record this along with the participant number and gender in an electronic secured access file.

The individual assignments will be available to the Adult ADHD team on a need to know basis. The local statistician/s will be independent and will not have access to the treatment allocations.

### Randomized treatments

#### Treatment as usual

All participants in the trial will receive TAU. This will include visits to their clinic doctor in the Service, if they have one, or visits to their local Specialist ADHD Service, CMHT or GP for management of their ADHD. They may be given some general advice but no specific advice with regard to strategies they may use to manage their ADHD symptoms. There will be no additional therapist involvement. The number of outpatient sessions will be recorded along with any treatments given to participants by the clinic doctor.

### CBT

In addition to treatment as usual, participants will also attend up to 15 weekly 50-minute CBT sessions over a period of 30 weeks as well as their treatment as usual sessions at the clinic. They will have a sixteenth, follow-up CBT session at 42 weeks.

During the early stages of treatment a shared formulation will be derived by patient and therapist. In the formulation links will be made between the individual’s predisposition, their early life experiences and current behavioural coping and cognitions. Treatment will then focus on the problematic cognitions and behaviours identified in the assessment and agreed in the formulation, while drawing on a range of evidence-based strategies such as thought records and behavioural experiments. Therapists will also have access to a range of skills-based approaches as have been used in previous studies, as and when they are indicated by the formulation (e.g. time management). A CBT manual has been developed but will be updated iteratively.

The aim will be to complete up to 15 sessions within 30 weeks. These will be scheduled approximately weekly several weeks ahead. If the patient is not able to attend an appointment because of ill health, the appointment will be rescheduled to within one week’s time. If it is not possible to rearrange the session within this timeframe, a session may be held over the phone. Ideally no more than three sessions in a row should take place over the phone. However if the choice is between not having a session and offering a telephone session, a telephone session will always be offered, even if three consecutive phone sessions have already taken place.

### Assessments

Wherever possible, assessments will be carried out at the adult ADHD Service. If the participant is unable to attend an appointment, an alternative appointment may be offered either at the participant’s convenience if not yet randomized, or within the two weeks of the post-assessment due date. If it is not possible for the participant to attend then questionnaires will be posted to them and the remainder of the assessment will be carried out over the phone.

Participants who drop out of treatment early will be assessed for outcomes as soon as possible rather than waiting for the normal follow-up.

It will not be practicable for the assistant psychologist to remain blind to the treatment group allocation and so we will not attempt this.

### Measures

See Table 1 for a summary of measures given by timepoint. We have chosen to measure both changes in symptoms and functional impairment as our primary outcomes.

**Table 1:** **Measures by time-point**

	**Completed by**	**Visit 1**	**30 weeks (end of therapy)**	**42 weeks (trial end)**	**Treatment discontinuation**	**Trial discontinuation**
**Eligibility assessment**	Clinic doctor/AP	X				
**Informed Consent to trial**	AP	X				
**Sociodemographic information**	AP	X				
**CAADID**	AP	X				
**MINI**	AP	X				
**Adult Barkley Current Behaviour Scale**	P	X	X	X	X	X
**Work and Social Adjustment Scale**	P	X	X	X	X	X
**Clinical Outcomes in Routine Evaluation Outcome measure (CORE-OM)**	P	X	X	X	X	
**Hospital Anxiety and Depression Scale (HADS)**	P	X	X	X	X	
**ADHD Cognitions Questionnaire**	P	X	X	X	X	
**ADHD Behaviours Questionnaire**	P	X	X	X	X	
**Rosenberg Self-Esteem Scale**	P	X	X	X	X	
**Autism Spectrum Quotient**	Only if not already completed	X				
**Frost Multidimensional Perfectionism Scale**	P	X	X	X	X	
**Beliefs about Emotions Questionnaire**	P	X	X	X	X	
**Adult Barkley Current Behaviour Scale – Other report form**	I	X	X	X	X	
**Global Impression scales -improvement**	IA, P, I, T		X	X	X	
**Global Impression scales -severity**	AP/IA	X (AP)	X (IA)	X (IA)	X (IA)	
**Global Impression scales -satisfaction**	P		X	X	X	
**Global Assessment of Functioning**	AP/IA	X (AP)	X (IA)	X (IA)	X (IA)	
**Rating of homework compliance**	T	Session by session				
**Ratings of treatment compliance, acceptance of model and therapeutic alliance**	T	X	X	X	X	
**Medications, dosage, adherence**	AP	X	X	X	X	
**Number of sessions TAU**	AP					

AP, Assistant psychologist; T, Therapist; P, Participant; I, Informant; IA, Independent assessor.

### Primary outcomes

#### Rated by participant (self-report)

1. Adult Barkley Current Symptoms Scale [30]
  The Barkley Current Symptoms Scale includes questions that assess the frequency of the 18 DSM–IV symptoms for ADHD. Items assess the frequency of 9 inattentive and 9 hyperactive/impulsive symptoms on a scale ranging from 0 = “never or rarely” to 3 = “often” with a score of 2 or above indicating the presence of a symptom. The presence of six or more symptoms for each subtype is considered clinically significant, in line with the recommended threshold of six out of nine symptoms in DSM- IV. This measure has strong reliability and validity and has been widely used in ADHD research.
2. Work and Social Adjustment Scale [31]
  This is a reliable and valid measure [32] that has been used widely to assess impairment in functioning in relation to an identified problem. It consists of 5 questions each rated on an 8-point scale (0 = “not at all impaired” and 8 = “very severely impaired”). A higher score indicates higher impairment.

### Secondary outcomes

#### Rated by participant (self-report)

3. Clinical Outcomes in Routine Evaluation Outcome measure (CORE-OM) [33]. This is a 34-item generic measure of psychological distress and is one of the most widely used outcomes for psychological therapies in primary and secondary care in the UK [34]. It has been shown to correlate well with other measures of psychological distress in an aggregated clinical sample and cut-off scores for determining clinical significance are available.
4. Hospital Anxiety and Depression Scale (HADS) [35]
  This is a 14-item scale with two subscales measuring anxiety and depression respectively. It has been found to reliably and validly assess the symptom severity and caseness of anxiety disorders and depression in psychiatric and primary care patients and in the general population [36].
5. ADHD Cognitions Questionnaire Participants will rate various beliefs in relation to their ADHD. The reliability and validity of this scale is being evaluated in a parallel study.
6. ADHD Behaviours Questionnaire Participants will rate the extent to which they engage in certain behaviours in relation to their ADHD. The reliability and validity of this scale is also being evaluated in a parallel study.
7. The Rosenberg Self-Esteem Scale [37].
  This is a 10-item uni-dimensional measure of global self esteem, self-worth or acceptance that has been shown to be reliable and valid including in psychiatric outpatients [38].
8. The Autism Spectrum Quotient [39] *†
  This is a 50-item questionnaire designed to assess autism spectrum symptoms in the general population. Results so far show good reliability. Studies in adults show that the scale can distinguish between ASD and controls as well as between ASD and psychiatric conditions such as OCD and social anxiety [39-42]. The scale’s ability to distinguish ASD from ADHD is, however, limited [43]. The scale is therefore only used as a screening tool in conjunction with the clinical assessment and the initial research assessment interview in assessing the presence of a possible autistic spectrum disorder. To the authors’ knowledge no alternative self-report scale of autistic spectrum disorder symptoms is available.
9. Global Impression scales (improvement** and satisfaction**) (adapted from Guy 1976 and used previously in Deale et al. 1997; [44,45]) provide self-rated global measures of improvement and treatment satisfaction on a seven point scale. Improvement is rated on a scale from 1 = “very much better” to 7 = “very much worse”; satisfaction is rated on a scale from 1 = “very satisfied” to 7 = “very dissatisfied”.
10. Frost Multidimensional Perfectionism Scale, Unhealthy Perfectionism subscale [46], The original scale had 35 items, however a subsequent factor analysis revealed two ‘higher order’ factors, interpreted as Healthy (Organisation and Personal standards;13 items) and Unhealthy perfectionism (Doubts about actions, Concerns over mistakes, Parental criticism, Parental expectations; 22 items) [47,48]. Responses are rated on 5-point Likert scale where 1 = “strongly disagree” and 5 = “strongly agree” and a higher score indicates greater perfectionism.
11. Beliefs about Emotions Questionnaire [49]
  This is a 12-item scale that assesses beliefs about experiencing or expressing negative thoughts and emotions. It was validated using participants with chronic fatigue syndrome and healthy controls and shows good internal reliability, validity and sensitivity to change. Responses are rated on a seven point Likert scale where 6 = “totally agree” and 0 = “Totally disagree”.

#### Nominated informant ratings

12. Adult Barkley Current Symptoms Scale – Other Report Form. This is an informant version of the Adult Barkley Current Symptoms Scale.
13. Global Impression Scales (improvement)**. Global measure of improvement (see 9) rated by the nominated informant.

#### Independent evaluator ratings

14. Global Impression scales (severity and improvement)**. Global measures of severity and improvement (See 9) rated by the independent evaluator.
15. Global Assessment of Functioning [50]. A generic rather than diagnosis-specific scoring system of psychological, social and occupational functioning and covers the range of positive mental health to severe psychopathology. For this study it will be used to provide a single score of functioning. While questions have been raised as to its utility for individual patients, it appears to have acceptable reliability at the group level [51].
16. The independent evaluator will also be asked to say which group they thought the participant was in and why (possible responses are “guess”, “the participant told me” and “other, please state”).

### Therapist ratings

17. Ratings of CBT compliance and adherence**. Number of CBT sessions attended, whether they were face-to-face or telephone, their duration and percentage completion of homework will be recorded. At the end of CBT treatment, the therapist will also score how well the participant adhered to the CBT approach, the extent to which the participant accepted the model of therapy and the therapeutic alliance on a five-point scale ranging from “not at all” to “completely”. They will also rate homework compliance on a scale of 0% to 100% completion on a session-by-session basis. These measures were adapted from a previous RCT [52]. A new measure was developed for the current study whereby the therapist will also rate their impression of the strength of the therapeutic alliance on seven point scale from 1 = “poor” to 7 = “excellent”.
18. Global Impression scales (severity and improvement) ** Global measures of severity and improvement rated by the therapist (See 9). For practical reasons these will be administered in the CBT condition only.

### Other

19. Medications and doses. At the each assessment point the assistant psychologist will take a record of current medications and doses, any changes, compliance with medication. They will also ask about any adverse events/reactions that may not yet have come to the attention of the trial personnel.
20. The details of TAU for all participants i.e. number of sessions with the service managing their ADHD (the Maudsley Adult ADHD Service or their local service) will be recorded.
  * baseline only.
  ** 30 weeks and 42 weeks only.
  † This is routinely administered during clinic assessments. This will only be included in the questionnaire pack if, for any reason, it has not already been completed.

### Ethics approval

The trial will be conducted in compliance with ethical approval (City Road and Hampstead Research Ethics Committee; reference 09/H0721/49) and R&D approval (reference R&D2009/051).

The study opened to recruitment February 2010.

### Sponsor

The main Sponsor is South London and Maudsley NHS Foundation Trust.

### Independent overseers

There will be a combined Trial Steering Committee (TSC) and Data monitoring and ethics committee (DMEC). They will be responsible for monitoring progress of the trial and serious adverse events or reactions. A trial management group will meet more regularly to discuss the day-to-day running and management of the trial. The combined TSC and DMEC will aim to meet at least three times over the course of the trial.

### Therapists’ compliance with treatment manual

Therapists’ compliance with treatment manuals will be monitored in two ways. All therapists will receive a minimum of monthly face to face group supervision sessions. All sessions will be audio-recorded and some recordings will be used to provide feedback to therapists on competence and treatment fidelity. Although the patient manual will be developed iteratively, the broad approach and content of therapy will be agreed by the principal investigator and the clinicians involved in the study (AD, TC, AR, KR) at the outset of the trial. Deviations from this will be noted and feedback given to the therapist. Following completion of the treatment sessions and of the patient and therapist manuals, 10% recorded sessions will be rated by two independent raters in order to assess adherence to the manual-defined therapy.

### Analyses

#### Sample size

The following power analysis is based on a previous study by Safren et al. [24] which compared CBT plus continued medication with continued medication only in ADHD and used the same primary outcome symptom measure as will be used in the current study.

A sample size of 23 in each group will have 90% power to detect a difference in means of 9.000. This is the difference between the change score of the Group 1 mean of 14.200 (SD = 7.2 assuming a correlation between pre and post measure of r = 0.5) and the Group 2 mean of 5.200 (SD = 9.05, assuming a correlation between pre and post of r = 0.5). This is also assuming that the common standard deviation is 9.050 (conservative estimate) using a two group *t*-test with a 0.050 two-sided significance level.

The intention to treat number is 23, however we will try to recruit 30 to each group to allow for dropouts.

#### Analysis plan

1. Between group effect sizes will be computed for the outcome measures.
2. The two conditions i) CBT and ii) TAU will be compared using random effects modelling. Intention to treat analysis will be used. It is predicted that the CBT group, covarying for baseline levels, will have better results than the TAU group on the primary outcome (i.e. lower score on self-rated ADHD symptoms and scores indicating higher levels of social and occupational functioning) and secondary outcome measures. A more detailed analytic structure will be written before data analysis begins.

## Discussion

This is a randomized controlled trial to further investigate the feasibility and acceptability of CBT in addition to TAU as compared with TAU alone. This is the first study to investigate individualized formulation-based CBT for adults with ADHD using a randomized controlled design. It is hoped it will provide important information about the efficacy of this approach in this client group. It will also provide information as to the feasibility of conducting a larger better-powered study in the future, potential effect sizes and acceptability of the approach to patients. Finally it will provide some preliminary information about mechanisms of change as well as predictors of successful CBT outcome.

A limitation of this study is that it is not designed to match for therapist time and attention and so it will not be possible to say whether any changes seen in the CBT group are specific to the CBT. Future studies should evaluate this formulation-based approach alongside an active control condition.

It is hoped this study will provide further information for people with ADHD, healthcare providers and commissioners as to which treatments are most useful for ADHD in adulthood. It will also provide some preliminary information about the mechanisms of change and predictors of outcome in terms of symptoms and functioning.
